# Tunable hyperbolic polaritons with plasmonic phase-change material In_3_SbTe_2_

**DOI:** 10.1515/nanoph-2023-0911

**Published:** 2024-02-13

**Authors:** Dunzhu Lu, Ying Zeng, Qizhi Yan, Qiyu Chen, Weiliang Ma, Xiao Luo, Ming Xu, Xiaosheng Yang, Peining Li

**Affiliations:** Wuhan National Laboratory for Optoelectronics and School of Optical and Electronic Information, Huazhong University of Science and Technology, Wuhan 430074, China; Optics Valley Laboratory, Hubei 430074, China; School of Integrated Circuits, Huazhong University of Science and Technology, Wuhan 430074 China; School of Information Engineering, Wuhan University of Technology, Wuhan 430070, China; National Engineering Research Center of Fiber Optic Sensing Technology and Networks, Wuhan University of Technology, Wuhan 430070, China; Hubei Key Laboratory of Broadband Wireless Communication and Sensor Networks, Wuhan University of Technology, Wuhan 430070, China

**Keywords:** hyperbolic polaritons, van der Waals materials, phase-change materials, In_3_SbTe_2_

## Abstract

Hyperbolic polaritons that originate from the extreme optical anisotropy in van der Waals (vdW) crystals have gained much attention for their potential in controlling nanolight. For practical use, there has been a strong interest to develop various manipulation strategies to customize the propagation of hyperbolic polaritons on a deeply sub-diffractional scale. In this regard, phase-change materials (PCMs) that possess two phases with different refractive indices offer suitably a tunable dielectric environment. Here, we report on the tuning of hyperbolic phonon polaritons in natural vdW crystals, hexagonal boron nitride (hBN), and alpha-phase molybdenum trioxide (α-MoO_3_), using the plasmonic phase-change material In_3_SbTe_2_ (IST). Unlike conventional PCMs whose both phases are dielectric, IST features a metallic crystalline phase that is stable at room temperature. The coupling between polaritons with their mirror charges in the underneath crystalline IST triggers an even stronger field confinement for polaritons. Moreover, benefited from the metallicity of laser-writable crystalline IST, we show an all-optical material platform in which crystalline IST boundaries efficiently excite and focus hyperbolic phonon polaritons in α-MoO_3_. Our experiments highlight the possibility to obtain new degrees of freedom in polariton engineering with plasmonic PCMs, thereby expanding the toolkit of tunable nanophotonics with flexible, on-demand fabrication and reconfiguration capabilities.

## 1 Introduction

Polaritons in natural van der Waals (vdW) materials provide vast opportunities for manipulating light on the nanoscale. This manipulation is achieved by exploiting their enhanced electromagnetic energy concentration, extreme anisotropic propagation, and strong inherent nonlinearities [1], [2], [3], [4], [5], [6], [7], [8], [9], [10]. One particular research focus lies with hyperbolic phonon polaritons (HPPs) originating from the coupling between photons and lattice vibrations. These phenomena occur in vdW materials with permittivities of opposite signs along different directions, e.g., hexagonal boron nitride (hBN) [4], [11], [12], [13] and alpha-phase molybdenum trioxide (α-MoO_3_) [1], [14], [15]. HPPs feature ultra-confined and highly directional light beam with hyperbolic dispersion, enabling novel nanophotonic applications such as hyperlensing [16], negative refraction [17], [18], molecular sensing [19], [20], [21], [22], and nanofocusing [23], [24].

In the ongoing quest for reconfigurable control of HPP properties [18], [25], [26], phase change materials (PCMs) emerge as an appealing platform. Their optical properties change significantly between the amorphous and crystalline phases subject to external stimuli, such as temperature control, laser pulses, or electrical bias [27], [28], [29]. As the electric field profile of polaritons is directly influenced by the dielectric environment, nonvolatilely switchable chalcogenide PCMs like Ge_3_Sb_2_Te_6_ have been often employed in a heterostructure with the hyperbolic material to actively tune HPPs [28]. However, the tuning range of HPP properties in this manner is limited by the permittivity contrast between the crystalline and amorphous phases of PCM. Moreover, to effectively launch HPP in the vdW/PCM heterostructure, an edge of the vdW material or a lithographically fabricated metallic structure is required [30], which makes the applications of polaritonic devices less flexible.

Here, we study the all-optical control of HPP propagation using “plasmonic PCM” In_3_SbTe_2_ (IST) [31], [32], [33], [34], [35], [36], [37], [38] in vdW/PCM heterostructures. IST has a crystalline phase (cIST) that features properties similar to noble metal, while its amorphous phase (aIST) remains dielectric, thus providing a significant optical contrast over infrared and terahertz spectral range [39]. By introducing an IST film in conjunction with hBN (α-MoO_3_) that support in-plane isotropic (anisotropic) HPPs, HPPs in the vdW material hybridize with their electromagnetic image charges in the metallic cIST [40], [41], [42], [43]. This hybrid polariton mode, referred to as hyperbolic “image polaritons” (HIPs) [42], [43], [44], not only enhances the field confinement but also provides reconfigurability of the polaritonic response. We also demonstrated that, with laser-switched crystalline microdomains in amorphous IST, the polariton propagation is conveniently manipulated in which polaritons can be effectively launched from a cIST boundary or focused by the end of a cIST antenna.

## 2 Results and discussion

**Figure 1:**
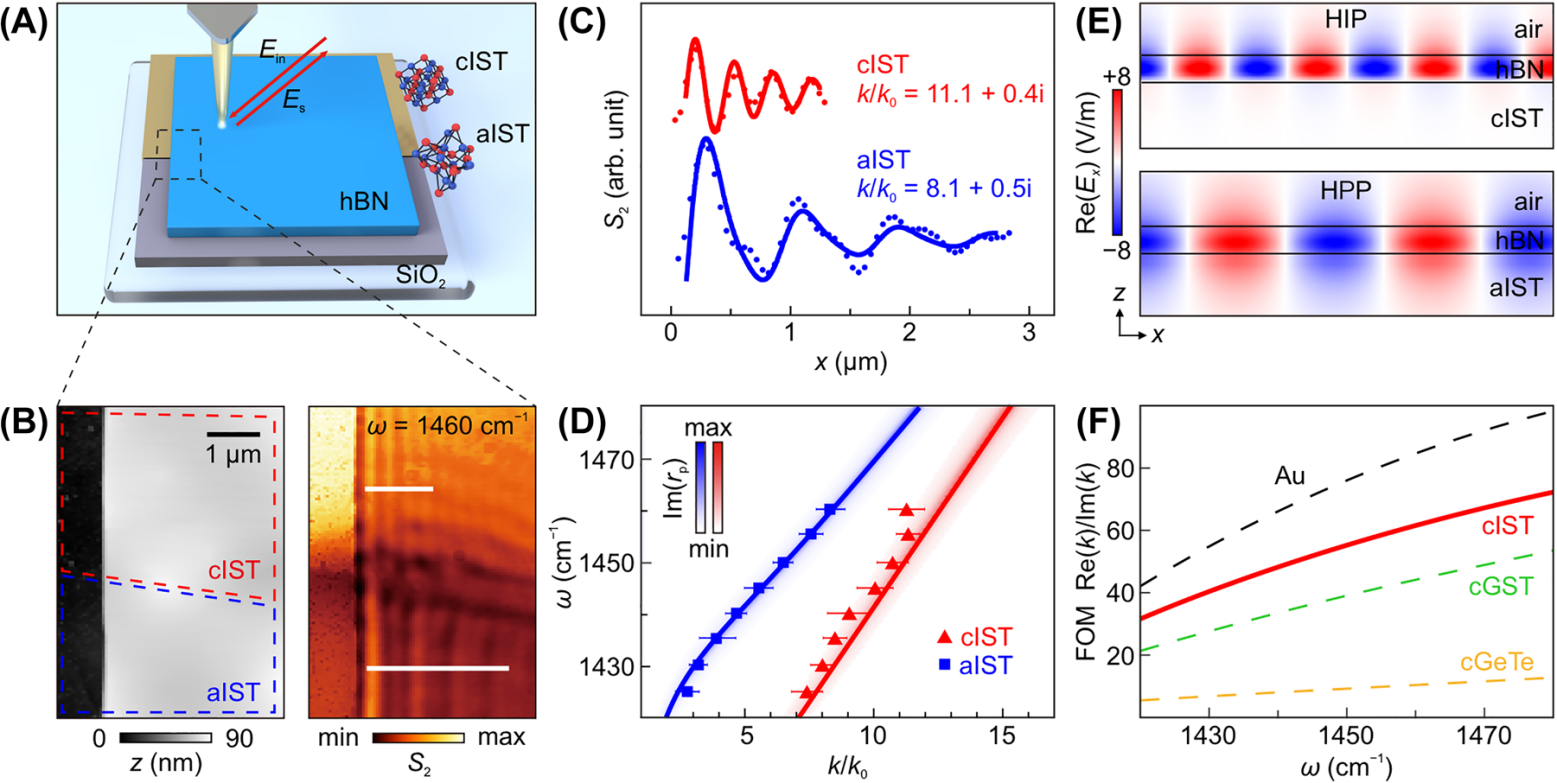
Near-field nanoimaging and dispersion analysis for phonon polaritons in hBN/IST heterostructure. (A) Schematic of s-SNOM nanoimaging experiment. (B) AFM topography image and near-field amplitude image (*ω* = 1460 cm^−1^) of the hBN (62 nm)/IST (80 nm) heterostructure. (C) Near-field amplitude profiles extracted along the white lines in (B) and corresponding fits. (D) Dispersion of phonon polaritons in hBN/cIST and hBN/aIST. False-color plot represents imaginary part of the Fresnel reflection coefficient, Im(*r*_p_), whose maxima are shown as solid lines. Triangles and squares are experimental data extracted from monochromatic s-SNOM measurements. (E) Cross-sectional electric field distribution Re(*E*_*x*_) of the HIP (HPP) mode propagating along hBN/cIST (hBN/aIST). (F) Theoretical FOM values for hBN/Au and different hBN/PCM heterostructures.

We first investigate the polariton propagation in a heterostructure that comprises an hBN layer (62 nm), a natural vdW material that supports in-plane isotropic HPPs, on an IST film (80 nm). In the latter, crystalline and amorphous phases coexist (Figure 1A, see Section 4). To visualize polaritons, we used a scattering-type scanning near-field optical microscope (s-SNOM). Under incident infrared field *E*_in_, the light is scattered from the atomic force microscope (AFM) tip of s-SNOM, thereby the polariton wavefront *E*_s_ is mapped as a function of tip position. Figure 1B shows the measured AFM topography image and near-field optical image of two adjacent regions of hBN/cIST and hBN/aIST. In the topography image, the dark and bright areas correspond to IST film and IST covered by hBN, respectively. While no significant difference in height is found between cIST (red dashed trapezoid) and aIST (blue dashed trapezoid) regions, the near-field image measured at 1460 cm^−1^ shows typical polariton fringe patterns of different periodicities on hBN/IST in these two regions.

To quantify the difference of propagating polaritons due to IST’s different phases in the heterostructure, we extracted near-field amplitude profiles along the white lines from Figure 1B and the results are shown in Figure 1C. Here, two different excitation sources for polaritons need to be considered [30]: the first is the hBN edge and the second is the AFM tip, which induces polariton interference fringes with spacing *λ*_p_ and *λ*_p_/2, respectively, where *λ*_p_ is the polariton wavelength. With this analytical model, we fit the data to find the complex wavevectors *k* of polaritons on hBN/cIST and hBN/aIST to be (11.1 + 0.4*i*)*k*_0_ and (8.1 + 0.5*i*)*k*_0_. The error bars are determined from the half width at half maximum (HWHM) obtained from the Fourier transform [45], [46]. These values indicate that the polaritons propagating on hBN/cIST exhibit larger field confinement compared to that on hBN/aIST. Meanwhile, the propagation loss of the former is slightly smaller judging from the imaginary parts. We note that polariton wavefronts observed at the boundary region between cIST and aIST are predominantly edge-excited. We also changed the illumination frequency between 1420 cm^−1^ and 1460 cm^−1^, namely within the type-II Reststrahlen band (1360–1610 cm^−1^) of hBN [4], and extracted the wavevector *k* of polaritons on hBN/cIST and hBN/aIST with the same approach from near-field images. The experimental data (squares and triangles in Figure 1D) are in good agreement with the analytically calculated dispersion (Section 4).

To understand the different polariton propagation constants on hBN/cIST and hBN/aIST heterostructures, we conducted numerical simulations (Section 4) for the electric field distribution across interface, see Figure 1E. With dielectric aIST, the electric field of HPP penetrates into both air and aIST. With metallic cIST, the presence of image charges in cIST induces the so-called HIP mode [44], in which the penetration of electric field into cIST is strongly suppressed and companied by a phase inversion. The metallicity of cIST is also evident when the figure of merit (FOM) of propagating polaritons, Re(*k*)/Im(*k*), is compared for heterostructures of hBN on various materials as a function of frequency (Figure 1F). Here, we have considered cIST, crystalline GST (cGST), crystalline GeTe (cGeTe), or gold as substrate for an 80-nm-thick hBN layer. Comparing the FOM values of those, cIST has the lowest relative loss among the different crystalline PCMs and demonstrate its potential as substitution for noble metals in infrared nanophotonics.

**Figure 2:**
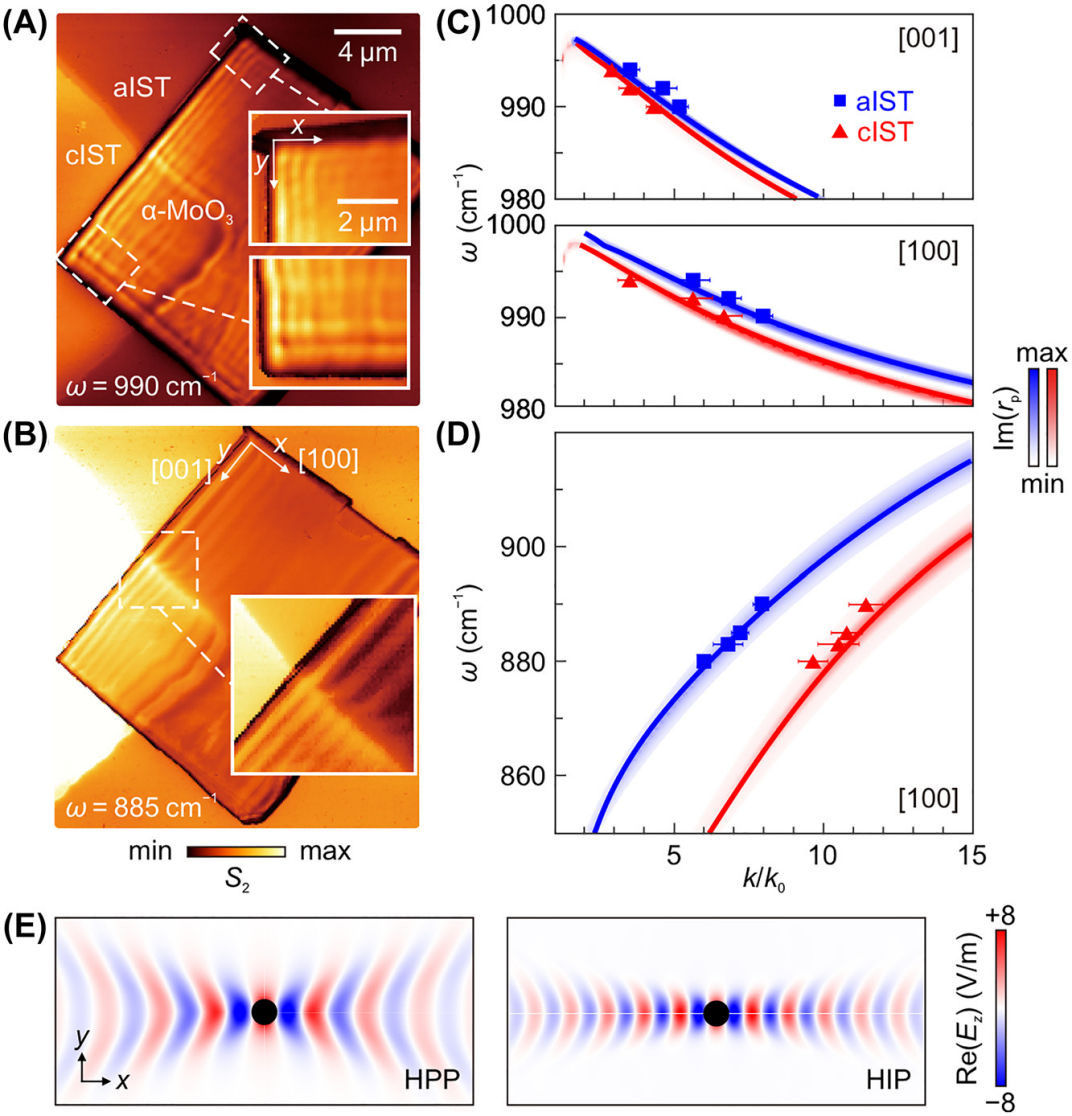
Near-field nanoimaging and dispersion analysis for phonon polaritons in α-MoO_3_/IST heterostructure. (A, B) Near-field amplitude image of the α-MoO_3_ (240 nm)/IST (80 nm) heterostructure, measured at (A) *ω* = 990 cm^−1^ and (B) *ω* = 885 cm^−1^. Insets show enlarged view of the dashed rectangular regions. (C, D) Dispersion of phonon polaritons in α-MoO_3_/IST within the (C) upper and (D) lower Reststrahlen bands. Labels on the top right corner mark the corresponding orientations of α-MoO_3_. False-color plot represents imaginary part of the Fresnel reflection coefficient, Im(*r*_p_), whose maxima are shown as solid lines. Triangles and squares are experimental data extracted from monochromatic s-SNOM measurements. (E) Simulated in-plane field distributions (*ω* = 885 cm^−1^) around an electric dipole source for HPP mode of α-MoO_3_/aIST and HIP mode of α-MoO_3_/cIST.

We now turn to the polariton propagation in an α-MoO_3_/IST heterostructure. Comparing with hBN that supports in-plane isotropic HPPs, α-MoO_3_ supports in-plane anisotropic HPPs with rich phonon modes and advantages of low loss and long lifetime. The in-plane dispersion of HPPs in α-MoO_3_ under the thin-film approximation [1] (*k*_0_ *d* ≪ 1, where *d* is the α-MoO_3_ thickness) can be analytically expressed as [47]:

(1) $$\begin{array}{c} k=\frac{\rho }{k_{0}d}\left[\arctan\left(\frac{\varepsilon_{1}\rho }{\varepsilon_{z}}\right)+\arctan\left(\frac{\varepsilon_{2}\rho }{\varepsilon_{z}}\right)+\pi l\right],\;\;\;l=0,1,2\ldots \end{array}$$

where $\rho =i\sqrt{\varepsilon_{z}/\left(\varepsilon_{x}\text{co}{\text{s}}^{2}\theta +\varepsilon_{y}\text{si}{\text{n}}^{2}\theta \right)}$ , *θ* is the angle between the *x* axis and the in-plane HPP wavevector *k*, *ε*_*x*,*y*,*z*_ is the permittivity of α-MoO_3_ along different orthogonal axis, and *ε*_1 _(*ε*_2_) is the permittivity of the media on top (at bottom) of the α-MoO_3_ film. We performed near-field nanoimaging experiment for a rectangle α-MoO_3_ flake with thickness *d* = 240 nm, under which both aIST and cIST regions are present. As shown in Figure 2A, the near-field amplitude image taken at 990 cm^−1^ shows periodic fringe patterns parallel to all flake edges along the [100] (*x* axis) and [001] (*y* axis) directions, as illustrated in the enlarged views (inset). In contrast, in Figure 2B, the image taken at 885 cm^−1^ shows similar fringe patterns exclusively parallel to the flake edge along the [001] direction. These results are in good agreement with previous reports on phonon polaritons of α-MoO_3_ exhibiting in-plane elliptic and hyperbolic dispersions in the upper (958–1010 cm^−1^) and lower (820–972 cm^−1^) Reststrahlen bands [1], respectively.

To quantitatively compare the difference of phonon propagation properties for α-MoO_3_/aIST and α-MoO_3_/cIST, we took multiple monochromatic s-SNOM images. Using the same analytical model for hBN that considers both the edge- and tip-excited modes, we extracted the wavevector values from line scans perpendicular to flake edges. The polariton dispersions for both cases and both Reststrahlen bands were plotted in Figure 2C and D, and an overall good agreement between theory and experiment is found. Notably, in the upper Reststrahlen band (Figure 2C), the in-plane anisotropy of phonon polaritons is reflected by dispersion curves of different slopes along the [100] and [001] directions. However, the difference of the in-plane polariton wavevector *k* for aIST and cIST cases are not pronounced. In the lower Reststrahlen band (Figure 2D), the polariton wavevectors supported by α-MoO_3_/aIST and α-MoO_3_/cIST heterostructures differ more, with the latter being clearly larger.

**Figure 3:**
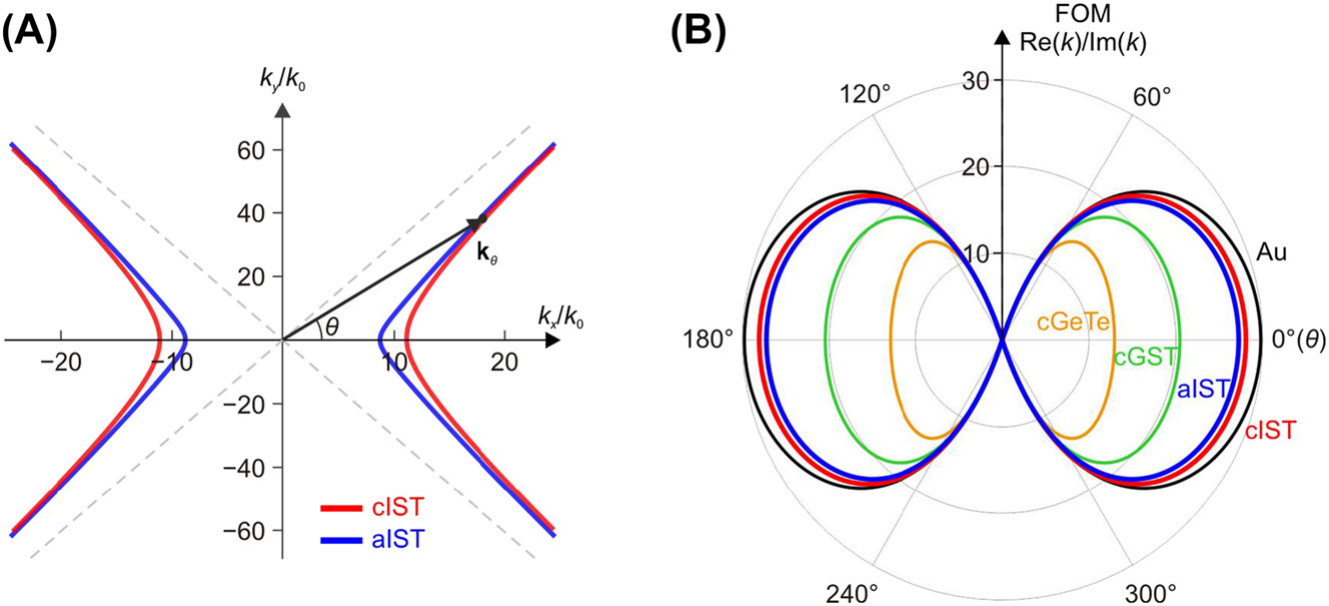
IFCs and FOM values of phonon polaritons for α-MoO_3_/IST. (A) IFCs of phonon polaritons for α-MoO_3_/cIST and α-MoO_3_/aIST. Dashed lines indicate the two asymptotes. Black arrow represents the wavevector of phonon polaritons with angle *θ* from the *x* axis of α-MoO_3_. (B) Comparing theoretical FOM values for α-MoO_3_/Au heterostructure and various α-MoO_3_/PCM heterostructures as a function of angle *θ*. Au and PCMs were modeled as infinite thick.

We also simulated the in-plane field distributions around an electric dipole source for α-MoO_3_/aIST and α-MoO_3_/cIST, see Figure 2E. The predicted wavefronts along the *x* axis for α-MoO_3_/cIST (Figure 2E, right panel) carries larger momentum than that for α-MoO_3_/aIST (Figure 2E, left panel), corresponding to the HIP and HPP mode in analogy to our analysis for hBN/cIST and hBN/aIST heterostructures (Figure 1E). The enhanced field confinement due to the presence of image charges in cIST is also evident in the calculated isofrequency contours for α-MoO_3_/cIST and α-MoO_3_/aIST at *ω* = 885 cm^−1^, see Figure 3A. Here, **k**_*θ*_ indicates an in-plane polariton wavevector with angle *θ* with respect to the *x* axis. When *θ* equals zero, the momentum difference of polaritons for α-MoO_3_/cIST and α-MoO_3_/aIST is maximized, with the former being approximately 1.3 times larger. As *θ* increases, this difference is gradually suppressed and both cases now enable a wavevector approaching infinity with an ultra-short wavelength. From a practical perspective, it is worth to compare the FOM values (Re(*k*)/Im(*k*)) of polaritons as a function of azimuthal angle for heterostructures of α-MoO_3_ on various PCMs (cIST, aIST, cGST, and cGeTe), as displayed by the polar plot in Figure 3B. For all types of PCM-based heterostructure calculated at *ω* = 885 cm^−1^, the phonon polaritons of α-MoO_3_ are available within the range between *θ* = ±60°. The α-MoO_3_/cIST heterostructure has overall largest FOM values for polaritons, except being marginally smaller than the α-MoO_3_/Au heterostructure.

**Figure 4:**
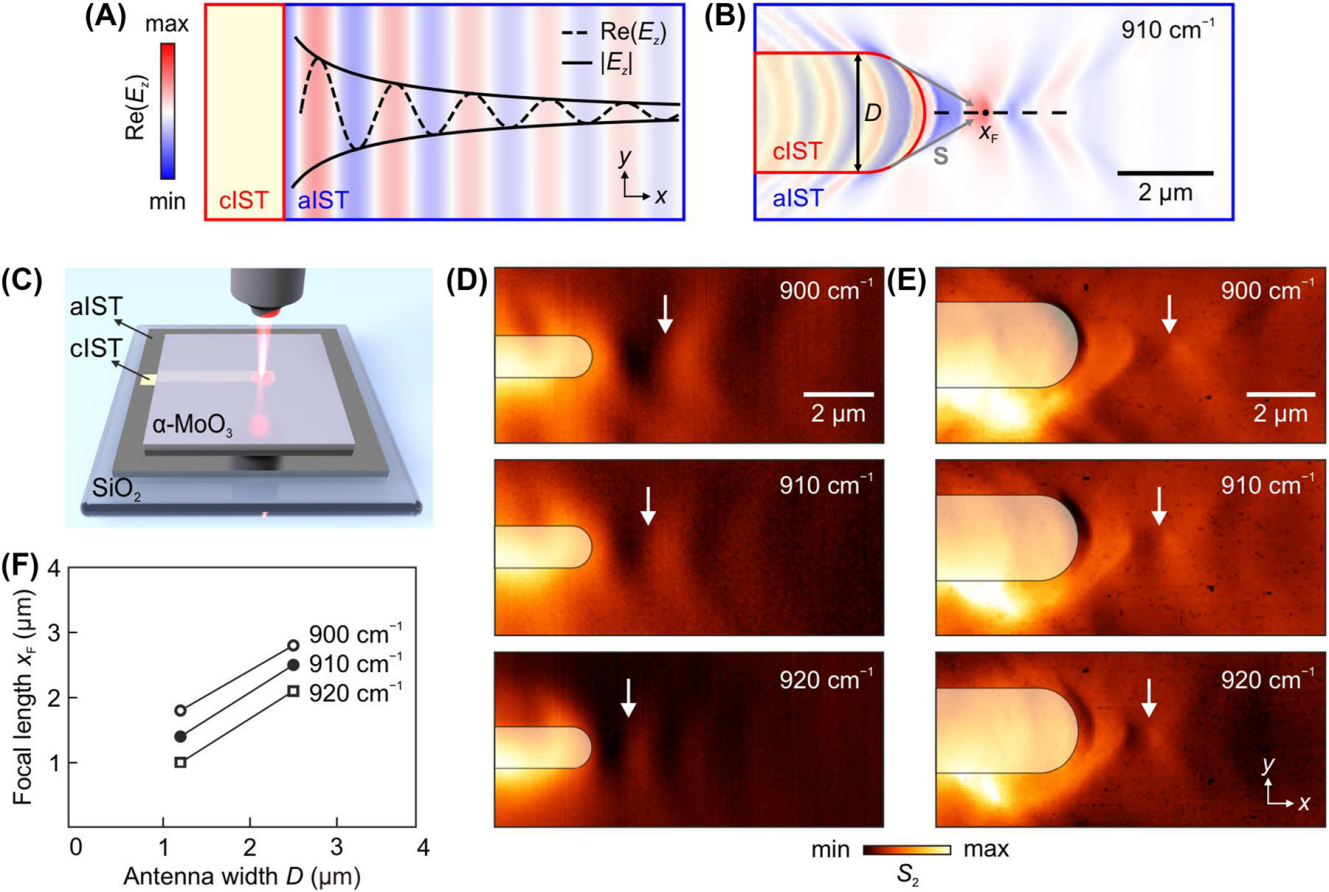
Launching and focusing HPPs with cIST boundary under α-MoO_3_. (A) Simulated electric field distribution of HPPs launched by a linear cIST boundary. (B) Simulated electric field distribution of HPPs focused by a rod antenna made of cIST. (C) Schematic of laser-switching the cIST rod antenna in an aIST film under α-MoO_3_. (D, E) Near-field amplitude images of the HPPs excited and focused by cIST rod antennas with widths (D) 1.2 μm and (E) 2.5 μm. Yellow-shaded areas represent where the crystallization of IST takes place. White arrows mark the foci positions. The illumination frequency was varied from 900 cm^−1^ to 920 cm^−1^ in experiment. The thicknesses of α-MoO_3_ and IST were 410 nm and 80 nm, respectively. (F) Focal lengths *x*_F_ extracted from the experimental near-field images.

Having established that phonon polaritons of different wavevectors can be excited from the flake edge of α-MoO_3_ on cIST or aIST, we further investigate polaritons that are launched by the boundary of cIST structures buried under α-MoO_3_. Figure 4A shows numerical simulations for polaritons launched from a linear boundary made of cIST, where beneath α-MoO_3_, the cIST and aIST regions are placed next to each other. The linear polariton wavefronts launched by the cIST edge are visible, whose amplitude gradually attenuates with increasing distance *x*. In contrast, with a rod antenna-like cIST, the polariton wavefronts on aIST are strongly deformed, see Figure 4B. Here, the interference of highly directional HPPs from different spatial position along the convex edge of cIST leads to the focus formation at a distance *x*_F_ from the antenna apex. This is similar to the case when a metallic (e.g., gold) disk is placed on top of α-MoO_3_ [23], but the advantage brought by our approach is that on-demand reconfiguration of HPPs is now possible with any thin hyperbolic medium on IST.

To experimentally validate our proposal, we transferred an α-MoO_3_ flake on an 80-nm-thick aIST film, in which rod antennas of cIST were subsequently laser-switched (Figure 4C, see Section 4). The orientation of the cIST antenna is aligned such that it is parallel to the [100] direction (*x* axis) of α-MoO_3_. In Figure 4D and E, cIST antennas of different widths, *D* = 1.2 μm and 2.5 μm, were imaged by s-SNOM at various illumination frequency. Here, yellow-dashed areas represent where the crystallization of IST takes place. Away from the cIST antenna’s right end, the edge-launched HPP wavefronts propagate along the *x* direction, first narrowing to a focal point and then diverging as moving forward. This behavior is in agreement with the numerical simulation shown in Figure 4B and resembles the free-space focusing by conventional convex lenses. Finally, in Figure 4F, the dependency of focal length *x*_F_ on the antenna width and illumination frequency is shown. As the antenna width increases from 1.2 μm to 2.5 μm, *x*_F_ extends by approximately 1 μm. As the illumination frequency increases from 900 cm^−1^ to 920 cm^−1^, which correspond to a wavelength decrease of 0.24 μm, *x*_F_ shrinks by 0.7–0.8 μm. We note that in Figure 4D and E, the brighter appearance of lower parts of antenna is due to an experimental artefact (see Figure S1). Our results show that both the size of phase-changed area as well as the incident infrared light can be utilized to fine-tune the planar subwavelength focusing of HPPs.

## 3 Conclusions

We have experimentally presented a reconfigurable heterostructure platform that uses the plasmonic PCM IST for tuning in-plane isotropic or anisotropic HPPs in hBN or α-MoO_3_. These results underscore the universality of IST in manipulating HPPs, not confined to a specific type of hyperbolic behavior. In particular, the metallicity of crystalline IST introduces a new avenue for controlling polariton properties. On one hand, the coupling between polaritons in the hyperbolic medium and their image charges in crystalline IST, known as the HIP mode, enhances the optical field compression of conventional HPP modes. On the other, we showcased the ability to launch polaritons from the edge of highly conductive crystalline IST, which provides an alternative to using the vdW crystal edge or a metal antenna as the polariton launcher. Furthermore, we demonstrated that a rod antenna made of crystalline IST with a convex boundary achieves the focusing of HPPs without the need for lithography. We anticipate that, as the dielectric-to-metal phase transition in IST is triggered by laser pulses in a nonvolatile fashion, microstructures of IST with arbitrary shapes [48] can be engineered for on-demand, customized manipulation and steering of polaritons.

## 4 Methods

### 4.1 Sample fabrication

In_3_SbTe_2_ (IST) was deposited on 2-mm-thick SiO_2_ substrates using stoichiometric targets by direct current magnetron sputtering (2 × 10^6^ mbar background pressure, 20 s.c.c.m. Ar flow, 0.15 nm/s deposition rate). The thickness of IST film was controlled by a stylus profiler (Brucker DektakXT) and the as-deposited IST films were in the amorphous state. Isotopically (10^B^) enriched hBN flakes were first prepared by the mechanical exfoliation using blue Nitto tape, before being transferred onto a polydimethylsiloxane stamp by a second exfoliation with the tape. We inspected the surface of the stamp under an optical microscope to identify the desired flake and transferred it onto an 80-nm-thick IST film on clean SiO_2_ substrates. α-MoO_3_ flakes were prepared and transferred with the same procedure.

### 4.2 Laser printing

A home-built setup was used to pattern the IST film by focusing a laser beam through a ×20 or ×50 objective (numerical aperture both 0.4) on the sample surface. A nanosecond laser diode source with a central wavelength of 660 nm provides single pulses with tunable output power up to 400 mW and pulse duration from 1 ns to 10 µs. To crystallize the IST film, laser pulses of 70 mW power and 80 ns duration were used. An *x*–*y* movable sample stage with maximum range of 50 mm in each direction and minimal step size of 700 nm was used to prepare the cIST areas (Figures 1B and 2A, B) and cIST antennas (Figure 4D and E). The scanning speed was set at 1–4 mms^−1^, while the repetition rate of the pulsed laser was not less than 3 kHz. While experimentally it is viable to write cIST after the transfer of vdW materials, for experiments in this work, the writing of cIST was performed first.

### 4.3 Near-field nanoimaging

For the infrared near-field nanoimaging, we employed a commercial s-SNOM system from Neaspec GmbH based on a tapping-mode AFM. The Pt-coated metallized tip (ArrowNCPt, NanoWorld) oscillates vertically with an amplitude of approximately 70 nm at a frequency Ω ≈ 270 kHz. The tip was illuminated by p-polarized light from a tunable continuous-wave quantum cascade laser. When the illuminated tip approaches the sample surface, the near-field interaction modifies the tip-scattered light, which is then recorded with a pseudo-heterodyne Michelson interferometer. To suppress background contribution induced from the tip shaft and distant sample, the detector signals are demodulated at a higher harmonic frequency *n*Ω (*n* ≥ 2), yielding the near-field optical amplitude *S*_*n*_ and phase *φ*_*n*_. For the near-field amplitude images shown in this manuscript, the second-order signal *S*_2_ was used.

### 4.4 Numerical simulations

Simulations were performed using finite-element method in the frequency domain with COMSOL Multiphysics. We used two-dimensional mode solver to calculate the electric field distribution across interface of hBN/IST heterostructures in Figure 1E. Wave optics module was used to obtain the in-plane field distributions around an electric dipole source and cIST rod antennas shown in Figures 2E and 4A, B, respectively. We used the transfer matrix method to calculate the eigenmodes of hyperbolic polaritons for various heterostructures. These eigenmodes are located in the poles of the Fresnel reflection coefficients for p-polarized light, *r*_pp_. We determine the poles by numerically solving the equation (1)/Abs(*r*_pp_) = 0. For spatially decaying modes, we considered real-valued frequencies and complex momenta. The dielectric permittivities of hBN, α-MoO_3_, and IST were modelled after references [15], [20], [32].

## Supplementary Material

Supplementary Material Details
