# Supplementary material for: Tunable hyperbolic polaritons with plasmonic phase-change material In3SbTe2
Source: Nanophotonics. 2024 Feb 13;13(6):937–44. doi: 10.1515/nanoph-2023-0911 (PMC11501199; doi:10.1515/nanoph-2023-0911)
Supplement: Supplementary file 1 — Supplementary Material Details [file j_nanoph-2023-0911_suppl_001.pdf]

Supplementary Material for

Tunable hyperbolic polaritons with plasmonic phase-change material In_3SbTe_2

Dunzhu Lu[1,2], Ying Zeng[3,4,5], Qizhi Yan[1,2], Qiyu Chen[1,2], Weiliang Ma[1,2], Xiao Luo[6], Ming Xu[6], Xiaosheng Yang[1,2*], Peining Li[1,2*]

1. Wuhan National Laboratory for Optoelectronics and School of Optical and Electronic Information, Huazhong University of Science and Technology, Wuhan 430074, China
2. Optics Valley Laboratory, Hubei 430074, China
3. School of information Engineering, Wuhan University of Technology, Wuhan 430070, China
4. National Engineering Research Center of Fiber Optic Sensing Technology and Networks, Wuhan University of Technology Wuhan 430070, China
5. Hubei Key Laboratory of Broadband Wireless Communication and Sensor Networks, Wuhan University of Technology, Wuhan 430070, China
6. School of Integrated Circuits, Huazhong University of Science and Technology, Wuhan 430074 China

*Corresponding author: yang_xs@hust.edu.cn, lipn@hust.edu.cn

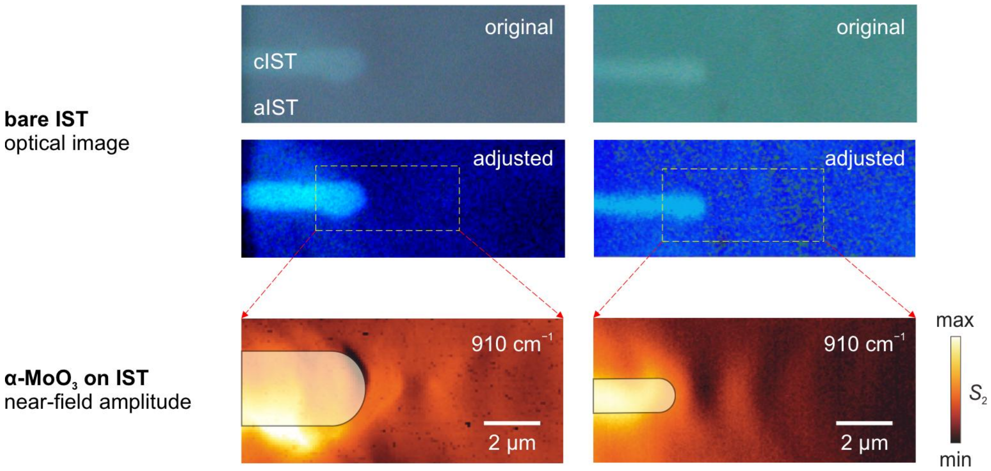

Figure S1. Optical images of cIST antennas (widths: 2.5 µm and 1.2 µm) measured with a conventional microscope and the corresponding experimental near-field amplitude (ω = 910 cm^{-1}) after the transfer of α-MoO_3. The apex of fabricated cIST antenna has some protrusion below that differs from the ideal geometry.
